# Conformational changes during human P2X7 receptor activation examined by structural modelling and cysteine-based cross-linking studies

**DOI:** 10.1007/s11302-016-9553-0

**Published:** 2016-12-26

**Authors:** Emily A Caseley, Stephen P Muench, Lin-Hua Jiang

**Affiliations:** School of Biomedical Sciences, Faculty of Biological Sciences, University of Leeds, Leeds, UK

**Keywords:** P2X7 receptor, Ion channel gating, Cysteine substitution, Disulphide bond, Cross linking

## Abstract

The P2X7 receptor (P2X7R) is important in mediating a range of physiological functions and pathologies associated with tissue damage and inflammation and represents an attractive therapeutic target. However, in terms of their structure-function relationships, the mammalian P2X7Rs remain poorly characterised compared to some of their other P2XR counterparts. In this study, combining cysteine-based cross-linking and whole-cell patch-clamp recording, we examined six pairs of residues (A44/I331, D48/I331, I58/F311, S60/L320, I75/P177 and K81/V304) located in different parts of the extracellular and transmembrane domains of the human P2X7R. These residues are predicted to undergo substantial movement during the transition of the receptor ion channel from the closed to the open state, predictions which are made based on structural homology models generated from the crystal structures of the zebrafish P2X4R. Our results provide evidence that among the six pairs of cysteine mutants, D48C/I133C and K81C/V304C formed disulphide bonds that impaired the channel gating to support the notion that such conformational changes, particularly those in the outer ends of the transmembrane domains, are critical for human P2X7R activation.

**Electronic supplementary material:**

The online version of this article (doi:10.1007/s11302-016-9553-0) contains supplementary material, which is available to authorized users.

## Introduction

P2X receptors (P2XR) are a structurally distinctive family of ligand-gated ion channels activated upon extracellular ATP binding, which are expressed in many cell types and play a role in a wide range of physiological functions [1–4]. These receptors are homo/hetero-trimers assembled from seven P2XR subunits (P2X1-7), which share a common structural arrangement comprising intracellular N- and C-termini and two transmembrane domains (TM1 and TM2) joined by a large extracellular domain. The crystal structure of the zebrafish P2X4 receptor (zfP2X4R) subunits with truncated N- and C-termini has been likened to the shape of a dolphin; the large extracellular domain constitutes the main body which is composed of the head, upper body, lower body, dorsal fin, left flipper and right flipper, and the two α-helical TM domains form the tail [5, 6]. The zfP2X4R structures in the closed and ATP-bound, open states provide the first structural insights into the non-conventional ATP-binding site and ion channel gating, in strong agreement with the results from site-directed mutagenesis studies of the mammalian P2XRs [3, 7]. Furthermore, the zfP2X4R structures have revealed extensive intra-subunit and inter-subunit interactions and considerable conformational changes within several parts, most noticeably the head and lower body of the extracellular domain and the two TM domains, during receptor activation [6].

The P2X7R, whilst exhibiting strong sequence similarity to other members of the P2X family and the conservation of key residues coordinating ATP binding, has unusual functional properties [8]. For example, P2X7R activation requires sub-millimolar concentrations of ATP, which is 10–100 times higher than that required for activation of the other P2XRs [9]. Activation of the P2X7R exclusively by high concentrations of ATP, which is found at the site of tissue damage or inflammation, renders the P2X7R to be considered as a ‘danger sensor’ [10]. It has been well documented that the P2X7R plays a critical role in both physiological responses to tissue damage and inflammation and the pathogenesis and progression of a diversity of associated conditions, including neuropathic and inflammatory pain, inflammatory diseases and cancers [11–15]. Evidently, detailed structural delineation of the P2X7R activation mechanism is important to better understand P2X7R-dependent physiological functions and diseases and also for the development of therapeutics targeting the human P2X7R (hP2X7R). Cysteine-based cross-linking, in combination with biochemical and functional assays, is a powerful approach to interrogate the interactions or relative movements between two adjacent parts of the same protein or between two neighbouring proteins [8]. This technique has been successfully applied in previous studies of ion channel gating (e.g. P2X2R [16], GABA_A_R [17] and K_V_ [18]), subunit stoichiometry of the P2X2/3R [19] and the inter-subunit ATP-binding site in the P2XR [20, 21] and in recent studies of agonist-induced conformational changes in the P2X1R [22], P2X2R [23] and P2X3R [24]. Here, we used this approach to examine conformational changes in the extracellular and transmembrane domains that are predicted, based on the structural models, to occur during the hP2X7R activation.

## Methods

### Homology modelling

Structural models of the hP2X7R were produced based on the structure of the zfP2X4R in the closed and ATP-bound open states (Protein Data Bank code 4DW0 and 4DW1, respectively) using Modeller version 9.12 [25] and analysed using MolProbity [26] as described in our previous studies [27, 28]. The non-conserved loop region between the β2 and β3 strands was modelled de novo using the ModLoop server [29]. These models are available from the authors on request. The wild-type (WT) hP2X7R sequence contained residues H155, R270 and A348.

### Site-directed mutagenesis

Single and double cysteine mutations were introduced into the plasmid encoding the hP2X7R using a PCR-based site-directed mutagenesis method [30]. In brief, the PCR sample of 50 μl contained 300 nM of each primer, 200 μM dNTP mix, 100 ng cDNA and 2.5 U PfuUltra DNA polymerase (Agilent). PCR consisted of the following steps: 96 °C for 60 s, 18 cycles consisting of 96 °C for 50 s, 60 °C for 50 s, 68 °C for 14 min and a final step of 68 °C for 30 min. The PCR product, after treatment with DpnI (Thermo Fisher Scientific) for 60 min, was transformed into competent *E. coli* cells (Stratagene). Small-scale isolation of plasmid was performed using a mini-DNA preparation kit (QIAGEN). Mutations were confirmed by commercial sequencing (Beckman Coulter Genomics).

### Cell culture and transient transfection

Human embryonic kidney (HEK) 293 cells were cultured in Dulbecco’s Modified Eagle Medium supplemented with 10% foetal bovine serum at 37 °C and 5% CO_2_, under humidified conditions. Cells were seeded in 6-well plates at 70–80% confluency prior to transfection and cells in each well were transfected using Lipofectamine2000 (Life Technologies) with 1 μg plasmid for the WT or mutant hP2X7R and 0.1 μg plasmid for enhanced green fluorescent protein (GFP), according to the manufacturer’s instructions.

### Whole-cell patch-clamp current recording

Cells were seeded onto 10-mm glass coverslips 20–24 h post transfection and single GFP-positive cells were chosen for recordings. Whole-cell currents were recorded at room temperature using an Axopatch 200B amplifier and analysed with pClamp 10.3 software (Axon instruments) as described in our previous studies [31, 32]. Cells were kept at a holding potential of −80 mV. BzATP and dithiothreitol (DTT) were applied using a RSC-160 rapid solution changer (Biologic Science Instruments). Patch microelectrodes with a resistance of 1–5 MΩ were produced using borosilicate glass capillaries (World Precision Instruments). Standard extracellular solution contained: 147 mM NaCl, 2 mM KCl, 1 mM MgCl_2_, 2 mM CaCl_2_, 10 mM HEPES and 13 mM glucose, pH 7.3. Intracellular solution contained 145 mM NaCl, 10 mM EDTA and 10 mM HEPES, pH 7.3. Divalent cations strongly inhibit the P2X7R and therefore BzATP-induced currents were mainly measured in low divalent extracellular solution containing 147 mM NaCl, 2 mM KCl, 0.3 mM CaCl_2_, 10 mM HEPES and 22 mM glucose, pH 7.3. Three hundred micrometer BzATP was repeated applied for 4 s every 2 min, and when the currents were fully facilitated, cells were exposed to 10 mM DTT between BzATP applications.

### Data analysis

All results, where appropriately, are presented as the mean ± standard error of mean (SEM). Statistical analysis was carried out using Student’s *t* test for two groups and one-way analysis of variance test and Tukey’s post hoc test for more than two groups, and the difference was considered to be significant at *p* < 0.05.

## Results

**Fig. 1:**
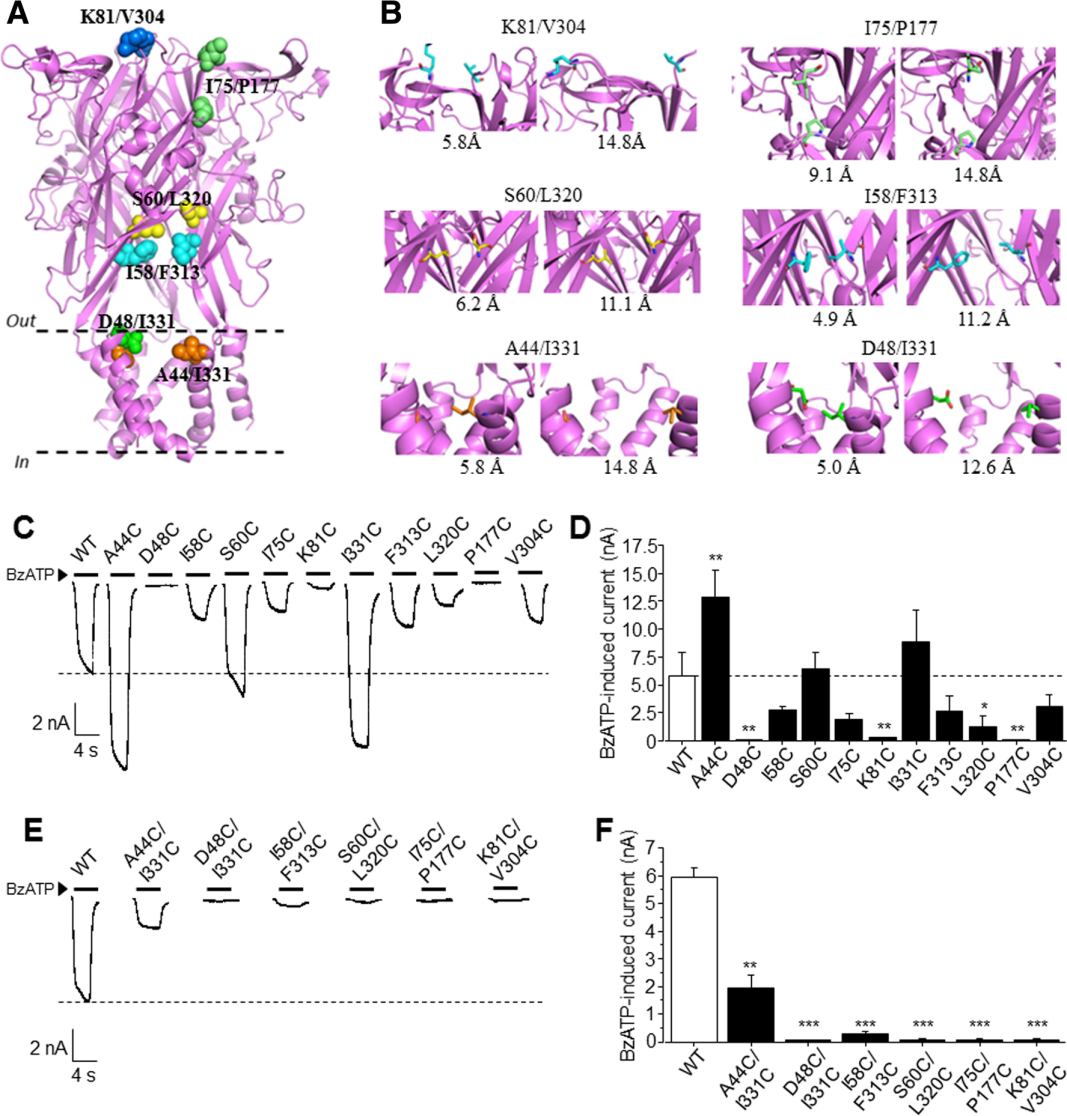
Location of residues in the hP2X7R targeted for cysteine substitution**. a** Homology model of the hP2X7R with pairs of residues that are subject to examination in this study coloured to correspond with *B*. **b** Expanded views of the pairs of residues indicated in *A* with sidechains indicated and distances between Cβ atoms of the identified pairs in the closed and open states. The closed state is shown on the *left panel* and the open state on the *right*. **c** Representative recording of whole-cell currents induced by 300 μM BzATP from HEK293 cells expressing the wild-type (WT) or indicated single mutant receptors. **d** Summary of BzATP-induced current amplitude for the WT or single mutant receptors. **e** Representative recording of whole-cell currents induced by 300 μM BzATP from HEK293 cells expressing the WT or indicated double mutant receptors. **f** Summary of BzATP-induced current amplitude for the WT or double mutant receptors. *p < 0.05; **p < 0.01; ***p < 0.005 compared to the WT receptor. Three to six cells were recorded in each case

Examination of the structural models of the hP2X7R in the closed and open states suggests that considerable conformational changes take place during receptor activation in the head, upper body and lower body of the extracellular domain as well as in the TM α-helices. Such conformational changes can be illustrated by the change in the distance, between the K81 and V304 (K81/V304) residues located in the upper body, I58/F311 and S60/L320 in the lower body, and A44/I331 and D48/I331 at the outer ends of the TM1 and TM2 domains (Fig. 1a, b). More specifically, these pairs of residues, contributed from two adjacent subunits, are predicted to be in the close vicinity to each other with the Cβ-Cβ distance in the range of 4.9–6.2 Å when the receptor is in the closed state, and move far apart, with the Cβ-Cβ distance increasing to 11.1–14.8 Å during the transition from the closed to open state (Fig. 1b). The I75/P177 in the head and from the same subunit is also expected to undergo substantial change with the Cβ-Cβ distance, changing from 9.1 Å in the closed state to 14.8 Å in the open state (Fig. 1b). These pairs of positions, if replaced with cysteines, may form disulphide bond in the closed state, but the large distance between these positions in the open state make the formation of disulphide bonds highly unlikely.

We introduced single and double cysteine substitutions into these six pairs of residues and performed whole-cell patch-clamp recordings to measure the currents in cells expressing individual single and double mutants. Currents were evoked by applying 300 μM BzATP, a super-maximal concentration at the WT receptor [32]. The single mutants bearing D48C, K81C or P177C were poorly functional, as evidenced by no or very small BzATP-induced current in cells expressing them (Fig. 1c, d). The other single mutants mediated noticeably variable BzATP-induced current responses; A44C mutation significantly increased, whereas L320C decreased, the amplitude of BzATP-induced currents as compared to that mediated by the WT receptor (Fig. 1c, d). In cells expressing the remaining five mutants, the current amplitude was moderately reduced (I58C, I75C, V304C and F313C) or increased (I331C), but the change was statistically insignificant. For the six double mutants, only expression of the A44C/I331C mutant led to considerable BzATP-induced currents and expression of the other double mutants gave rise to very small but discernible BzATP-induced currents (Fig. 1e, f). None of the single or double mutations altered the holding current.

**Fig. 2:**
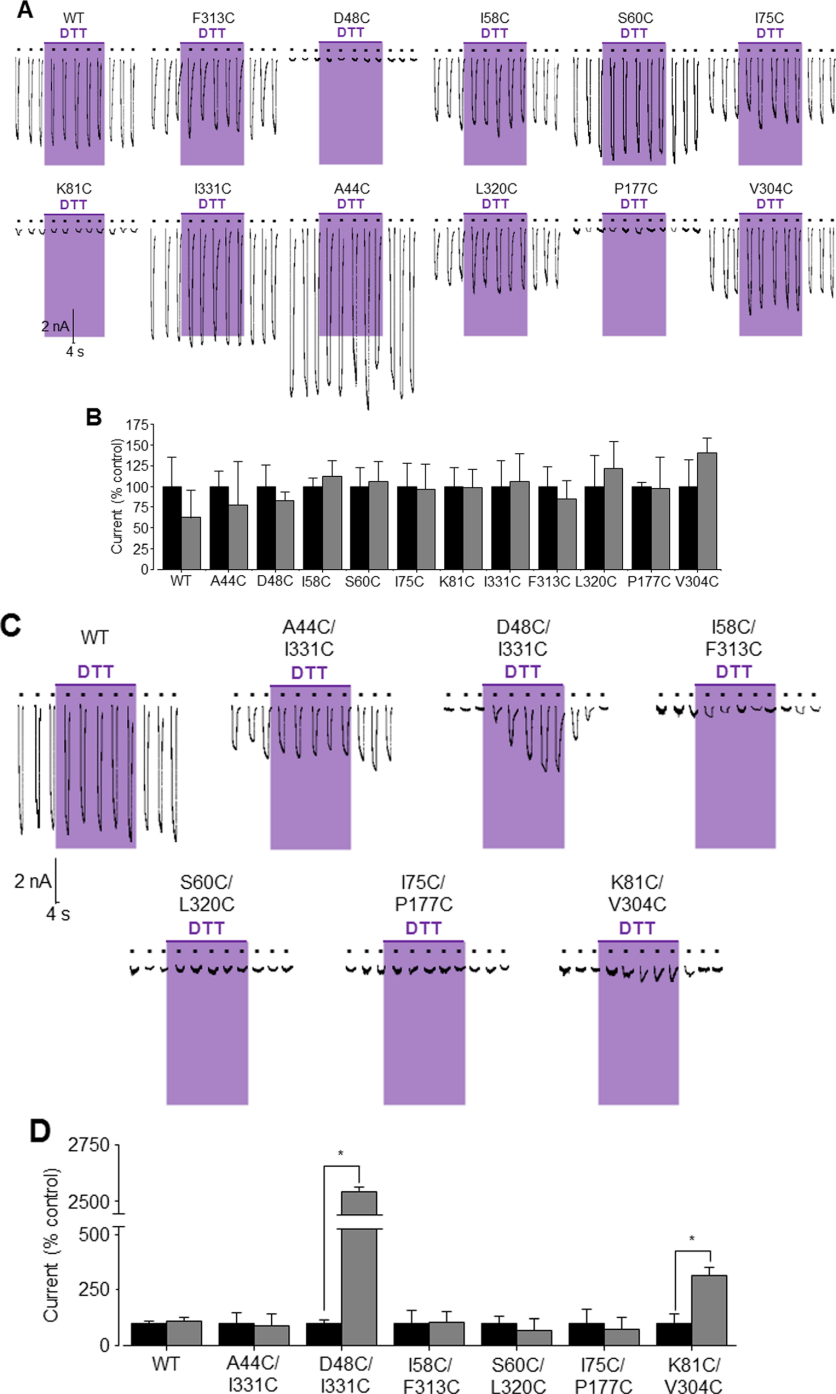
Effects of treatment with DTT on BzATP-induced currents mediated by the WT and single and double cysteine mutant hP2X7Rs. **a** Representative whole-cell recordings showing BzATP-induced currents prior to, during and after exposure to 10 mM DTT in HEK293 cells expressing the WT or indicated single mutant receptors. **b** Summary of the effects of DTT treatment on the WT or single mutant receptors by expressing BzATP-induced currents before and at the end of 10-min exposure to DTT as percentage of the mean currents immediately before exposure to DTT. The *grey* and *black columns* represent the mean currents in percentage before and 10 min after DTT exposure, respectively. **c** Representative whole-cell recordings showing BzATP-induced currents prior to, during and after exposure to 10 mM DTT in HEK293 cells expressing the WT or indicated double mutant receptors. **d** Summary of the effects of DTT treatment on the WT or indicated mutant receptors by expressing BzATP-induced currents at the end of 10-min exposure to DTT as a percentage of the mean currents immediately before exposure to DTT. The *grey* and *black columns* represent the mean currents in percentage pre- and post-DTT application, respectively. *p < 0.05. Three to six cells were recorded for each case

We moved on to compare BzATP-induced currents in cell expressing the WT or mutant receptor before and after exposing the patched cells to DTT, a reducing agent. Exposure to DTT for 10 min was without effect on the holding currents (data not shown) and also had no effect on BzATP-induced currents mediated by the WT receptor or single mutants (Fig. 2a, b). Lack of significant effects of DTT on BzATP-induced currents were also observed in cells expressing the A44C/I331C double mutant or the poorly functional I58C/F313C, S60C/L320C and I75C/P177C double mutants (Fig. 2c, d). In striking contrast, treatment with DTT caused a progressive and remarkable increase in small current BzATP-induced currents in cells expressing the D48C/I331C double mutant. The current amplitude in the steady state after DTT treatment reached 64 ± 12% of that mediated by the WT receptor (Fig. 2c). In addition, the effect of DTT on D48C/I331C mutant-mediated currents was readily reversed upon washing (Fig. 2c). Treatment with DTT also significantly enhanced BzATP-induced currents in cells expressing the K81C/V304C double mutant, albeit the current amplitude still remaining much smaller relative to that mediated by the WT receptor (Fig. 2c, d).

## Discussion

As introduced above, the P2X7R is physiologically and therapeutically important but our current understanding regarding its activation and the conformational changes which accommodate this has been mainly inferred by structural homology modelling and studies of single nucleotide polymorphic mutations [27]. In this study, by combining cysteine-based cross-linking with patch-clamp recording, we probed conformational changes in the head, upper and lower body of the large extracellular domain and the outer ends of the transmembrane domains associated with hP2X7R activation. Specifically, we examined six pairs of residues located in these parts which are predicted by structural models to undergo considerable movement during the transition of the ion channel from the closed to open state (Fig. 1a, b). These 11 residues are present in mammalian P2X7Rs but not conserved among the P2X receptor family, with an exception of residues at three positions 75, 81 and 304 [1, 6, 27], and many of them are also different from those in the rP2X2R examined in a recent study [23] (supplemental Fig. 1).

Introduction of single or double cysteine substitutions predominantly impaired or ablated receptor function (Fig. 1c, d). Exposure to DTT did not rescue any single mutant with severely impaired function (Fig. 2a, b). These results suggest that cysteine substitution of the residues under investigation introduced deficiencies in protein synthesis, membrane trafficking, activation or a combination of these, which requires further study for clarification. Such deficiencies are also likely responsible for further loss of function in the double mutants including I58C/F313C, S60C/L320C, I75C/P177C and K81C/V304C. Notably, the amplitude of BzATP-induced currents mediated by the A44C/I331C double mutant was significantly reduced compared to that mediated by the WT receptor (Fig. 1e, f). This result was not anticipated, considering that the A44C and I331C mutations both individually increased the current amplitude (Fig. 1d). The currents mediated by the A44C/I331C double mutant were insensitive to DTT (Fig. 2c, d), largely excluding the possibility that formation of disulphide bond hinders the ion channel opening as discussed further below. Currently, we cannot offer any straightforward interpretation.

The most interesting finding in this study is the contrast in results from the D48C/I331C and A44C/I331C double mutants. BzATP induced very small but discernible currents in cells expressing the D48C/I331C mutant (Fig. 1e, f). However, BzATP-induced currents increased progressively during treatment with DTT and in the steady state reached more than half of that mediated by the WT receptor (Fig. 2c). Furthermore, DTT-induced effect was readily reversed by washing (Fig. 2c). These results were not observed for BzATP-induced currents mediated by the single mutants (Fig. 2a, b). The most plausible interpretation is that D48C and I331C form an inter-subunit disulphide bond in the closed state and such cross-linking is sufficient to lock the receptor ion channel in the closed state, as described in previous studies for the rP2X2R and rP2X2/3R [16, 19, 23]. The Cβ-Cβ distance between D48 and I331 is 5.0 and 12.6 Å in the closed and open states, respectively, suggesting that these two residues move a considerable distance from each other as the hP2X7R ion channel switches from the closed to open state. Our results clearly support the existence of such conformational changes at the outer ends of the TM helices. In contrast with the D48C/I331C double mutant, expression of the A44C/I331C double mutant resulted in considerable BzATP-induced currents (Fig. 1e, f), and A44C/I331C mutant-mediated currents were completely insensitive to DTT (Fig. 2c). The Cβ-Cβ distance between A44 and I331 is predicted to be 5.8 Å in the closed state (Fig. 1b), which is similar to that between D48 and I331. One possible explanation is therefore that A44C and I331C form a disulphide bond but the disulphide bond does not hinder receptor activation. This explanation is clearly not appealing, because A44 and I331 are predicted to fall 14.8 Å apart in the open state (Fig. 1b), which is too great a distance to retain the disulphide bond between A44C and I331C. A44 is located approximately one α-helix underneath D48 in the TM1 domain (Fig. 1a). Such an explanation is also hard to reconcile with the considerable conformational change at the outer ends of the two TM domains as indicated by the results from the D48C/ I331C double mutant. An alternative, more likely, explanation is that A44C does not form a disulphide bond with I331C. This explanation is more consistent with the structural models, in which A44 is predicted to be nestled more deeply in the TM1 domain and not well positioned towards I331 (Fig. 1b). Overall, our results from examining these two double mutants, particularly D48C/I331C, provide clear evidence to support the conformational changes in the outer ends of the TM domains accompanying hP2X7R activation.

The upper body, based on comparison of the zfP2X4R structures in the closed and open states, remains much less mobile than other parts of the extracellular domain during receptor activation. In this study, we examined K81 and V304 in the upper body of the hP2X7R which are predicted to move significantly away from each other during receptor activation (Fig. 1a, b). The K81C mutation impaired the receptor function (Fig. 1c, d), which also accounts for the poor functional expression of the K81C/V304C double mutant (Fig. 1e, f). Treatment with DTT had no effect on BzATP-induced currents in cells expressing the K81C or V304C single mutants (Fig. 2a, b), but significantly enhanced BzATP-induced currents in cells expressing the K81C/V304C double mutant, although the current remained very small (Fig. 2c, d). These results suggest the formation of a disulphide bond between K81C and V304C in the receptors that were trafficked to the cell surface. These results also support the increased separation between K81 and V304 in the upper body predicted by the structural models (Fig. 1b) when the receptor switches from the closed to the open state.

In summary, our study provides evidence to support the notion that conformational changes in the upper part of the extracellular and the outer ends of the transmembrane domains are crucial for hP2X7R activation.

## Electronic supplementary material

ESM 1 (PDF 143 kb)
