# Supplementary material for: Conformational changes during human P2X7 receptor activation examined by structural modelling and cysteine-based cross-linking studies
Source: Purinergic Signal. 2016 Dec 26;13(1):135–41. doi: 10.1007/s11302-016-9553-0 (PMC5334206; doi:10.1007/s11302-016-9553-0)
Supplement: Supplementary file 1 — (PDF 143 kb) [file 11302_2016_9553_MOESM1_ESM.pdf]

**Supplemental data**

# Conformational changes during human P2X7 receptor activation examined by structural modelling and cysteine-based cross-linking studies

Emily A Caseley, Stephen P Muench and Lin-Hua Jiang*

School of Biomedical Sciences, Faculty of Biological Sciences, University of Leeds, U.K.

*Correspondence to: Lin-Hua Jiang (E-mail: l.h.jiang@leeds.ac.uk).

**Fig. S1 Cysteine-based crossing-linking studies of residues in P2X receptors**

Amino acid sequence alignment of the extracellular domain in human P2X1, rat P2X2, human P2X3 and human P2X7 receptors. The 10 conserved cysteine residues are in yellow background and the pair of cysteines participate in formation of the same disulphide bond are indicated with the same symbols on top of cysteine residues. The residues, which were examined with cysteine substitution in the present and previous studies, are in green background, and the pair of residues, if both replaced with cysteine, can form disulphide bond are indicated by the same symbols on top of residues. Note that cysteine introduced in some positions can disulphide bond with cysteine in more than one positions.

```
                       ◊ ◀
hP2X1    WVFLYEKGYQTSS-GLISSVSVKLKGLAVT-------QLPGLGPQVWDVADYVFPAQGDN   98
                           ~
          @  >  <          #                                   :*
rP2X2    YVFIVQKSYQDSETGPESSIITKVKGITMS------------EDKVWDVEEYVKPPEGGS   94
                         =
hP2X3    WVFLHEKAYQVRDTAIESSVVTKVKGSGLY------------ANRVMDVSDYVTPPQGTS   88
              ♠                                 ♣
hP2X7    FALVSDKLYQRKE-PVISSVHTKVKGIAEVKEEIVENGVKKLVHSVFDTADYTFPLQG-N  100

                           ■          ▲     ●     △          ▲
hP2X1    SFVVMTNFIVTPKQTQGYCAEHPE--GGICKEDSGCTPGKAKRKAQGIRTGKCVAFNDTV  156
           :
           *
rP2X2    VVSIITRIEVTPSQTLGTCPESMRVHSSTCHSDDDCIAGQLDMQGNGIRTGHCVPYYHGD  154
                                  +
hP2X3    VFVIITKMIVTENQMQGFCPESE--EKYRCVSDSQC--GPERLPGGGILTGRCVN-YSSV  143

hP2X7    SFFVMTNFLKTEGQEQRLCPEYPTR-RTLCSSDRGCKKGWMDPQSKGIQTGRCVVHEGNQ  159

                                                 ⌂
            ●     ■               △        ○     ▶▼
hP2X1    K-TCEIFGWCPVEVDDDIPRPALLREAENFTLFIKNSISFPRFKVNRRNLVEEVNAAHMK  215
                                             #
rP2X2    SKTCEVSAWCPVEDGTSDNHFLGK-MAPNFTILIKNSIHYPKFKFSKGN-IASQKSDYLK  212
                                                                 +
hP2X3    LRTCEIQGWCPTEVDT-VETPIMM-EAENFTIFIKNSIRFPLFNFEKGNLLPNLTARDMK  201

hP2X7    K-TCEVSAWCPIEAVEEAPRPALLNSAENFTVLIKNNIDFPGHNYTTRNILPGLNIT---  215

          ♦         ♦                                 □        □
hP2X1    TCLFHKTLHPLCPVFQLGYVVQESGQNFSTLAEKGGVVGITIDWHCDLDWHVRHCRPIYE  275

rP2X2    HCTFDQDSDPYCPIFRLGFIVEKAGENFTELAHKGGVIGVIINWNCDLDLSESECNPKYS  272

hP2X3    TCRFHPDKDPFCPILRVGDVVKFAGQDFAKLARTGGVLGIKIGWVCDLDKAWDQCIPKYS  261

hP2X7    -CTFHKTQNPQCPIFRLGDIFRETGDNFSDVAIQGGIMGIEIYWDCNLDRWFHHCRPKYS  274

                                                          ▶
                                                          ◀ ▼
                     ○                                    ◊ ⌂
hP2X1     FHGLYE---EKNLSPGFNFRFARHFVEN-GTNYRHLFKVFGIRFDILVDGKAGKFDIIP  330
                                                                   <
                                                                   >
                                                      ~            @
rP2X2     FRRLD--PKYDPASSGYNFRFAKYYKIN-GTTTRTLIKAYGIRIDVIVHGQAGKFSLIP  329
           =
hP2X3     FTRLDSVSEKSSVSPGYNFRFAKYYKMENGSEYRTLLKAFGIRFDVLVYGNAGKFNIIP  320
                                        ♣                          ♠
hP2X7     FRRLDDKTTNVSLYPGYNFRYAKYYKE-NNVEKRTLIKVFGIRFDILVFGTGGKFDIIQ  332
```
